# QuantSeq. 3′ Sequencing combined with Salmon provides a fast, reliable approach for high throughput RNA expression analysis

**DOI:** 10.1038/s41598-019-55434-x

**Published:** 2019-12-11

**Authors:** Susan M. Corley, Niamh M. Troy, Anthony Bosco, Marc R. Wilkins

**Affiliations:** 1 Systems Biology Initiative, School of Biotechnology and Biomolecular Sciences, UNSW Sydney, New South Wales, Australia; 2 Telethon Kids Institute Australia, The University of Western Australia, Perth, Australia

**Keywords:** Computational biology and bioinformatics, Gene expression

## Abstract

RNA-Seq is increasingly used for the diagnosis of patients, targeting of therapies and for single cell transcriptomics. These applications require cost effective, fast and reliable ways of capturing and analyzing gene expression data. Here we compared Lexogen’s QuantSeq which captures only the 3′ end of RNA transcripts and Illumina’s TruSeq, using both Tophat2 and Salmon for gene quantification. We also compared these results to microarray. This analysis was performed on peripheral blood mononuclear cells stimulated with Poly (I:C), a viral mimic that induces innate antiviral responses. This provides a well-established model to determine if RNA-Seq and QuantSeq identify the same biological signatures. Gene expression levels in QuantSeq and RNA-Seq were strongly correlated (Spearman’s rho ~0.8), Salmon and Tophat2 (Spearman’s rho > 0.9). There was high consistency in protein coding genes, non-concordant genes had a high proportion of shorter, non-coding features. RNA-Seq identified more differentially expressed genes than QuantSeq, both methods outperformed microarray. The same key biological signals emerged in each of these approaches. We conclude that QuantSeq, coupled with a fast quantification method such as Salmon, should provide a viable alternative to traditional RNA-Seq in many applications and may be of particular value in the study of the 3′UTR region of mRNA.

## Introduction

Changes in gene expression reflect changes in cellular function and behaviour, in development and in disease states. Over the past two decades, methods of measuring gene expression have improved dramatically with a plethora of hybridization arrays available followed by RNA-Seq, the sequencing of short or long RNA reads using massively parallel sequencing technology. There is now a wealth of scientific literature detailing new analysis methods and describing the applications of these approaches^1–4^. Transcriptomics research has matured to the extent that its benefits for clinical diagnosis and treatment of patients can be explored^5^. Already, research is being done to utilize RNA-Seq in a clinical setting to detect early stage head and neck cancer^6^, to inform treatment options in breast cancer^7,8^, in predicting adverse immune responses in organ transplant cases^9^ and in cancer immunotherapy^10^. The advent of single cell RNA-Seq, involving the simultaneously sequencing of RNA from thousands of individual cells^11^, brings additional new potential for understanding the mechanisms of disease. Moving from a research environment to a clinical environment has many additional challenges. Results need to be obtained quickly, so that patient diagnosis and care is not unreasonably delayed. Sample volume may be high and cost to the health system needs to be minimised. How best to do this are important questions at this time.

QuantSeq. 3′ mRNA library preparation predominantly produces fragments for sequencing close to the 3′ end of polyadenylated mRNA, generally from the last exon and the 3′ untranslated region (3 UTR)^12^. The QuantSeq method uses total RNA as input, there is no prior poly(A) enrichment or rRNA depletion. The QuantSeq Forward kit has an oligo (dT) primer which contains the Illumina-specific Read 2 linker (P7), which is annealed to the 3′ end of the mRNA fragment to synthesise the first cDNA strand via reverse transcriptase. The second strand synthesis is commenced by random priming and DNA polymerase extension. The random primer contains the Illumina-specific Read 1 linker sequence (P5). Sequencing commences from the Read 1 sequencing primer and goes toward the poly(A) tail with only one fragment produced per transcript. QuantSeq therefore differs from traditional RNA-Seq where multiple reads are generated from any part of the RNA transcript.

As QuantSeq sequences a smaller part of the transcript and produces only one read per transcript, less sequencing should be required than for standard RNA-Seq. The QuantSeq vendor, Lexogen, recommends that 10 M reads per sample are required for QuantSeq mammalian transcriptomics. In addition to requiring less sequencing, the 3′ sequencing method was originally conceived as a way of avoiding gene length bias seen in RNA-Seq^13^. Several studies have investigated the effect of sequencing depth on RNA-Seq and whilst higher power is achieved as sequencing depth increases, beyond 20–30 M reads the gain in power is minimal^14,15^. Nevertheless, ideal read depth will depend on the goals of the experiment, for example the GTEx project used 50 M reads per sample to assess allele-specific expression^16^.

Salmon^17^ like kallisto^18^, is a new quantification method which avoids direct base-by-base mapping to the genome, such as occurs with Tophat2^19^, the newer version HISAT2^20^ or STAR^21^, and instead quantifies transcripts through quasi-mapping and probabilistic modelling of transcript abundance. To commence quantification the user provides a transcripts file (fasta format) as input and reads are assigned to these transcripts. The output of Salmon is abundance per transcript. The R package Tximport^22^ can be used and was used here to summarise transcript abundance to the gene level. The motivation for using QuantSeq and Salmon is similar, with both potentially providing a straightforward means of gene expression analysis. This may be of particular benefit in emerging clinical applications as well as for single cell RNA-Seq which by its very nature involves transcript quantification of a very large number of samples, although at lower number of reads per sample.

Here we compare Illumina’s TruSeq RNA-Seq and the Lexogen QuantSeq, and also compare the results from use of read mapping by TopHat2 and Salmon. It is of course most important that any new approach gives reliable informative results. We have therefore used a test case in a well-established model involving peripheral blood mononuclear cells (PBMCs) stimulated with Polyinosinic-polycytidylic acid (Poly (I:C)), a potent inducer of anti-viral responses. Poly(I:C) activates the viral RNA-sensing pattern recognition receptors (PRRs) TLR3, RIG-I/MDA5 and PKR. Activation of these PRRs induces a series of intracellular signalling cascades that trigger nuclear translocation of transcription factors NF-kB and IRF family, leading to cellular expression of pro-inflammatory and antiviral genes^23,24^. Using this model we expect to see changes in gene expression consistent with the innate anti-viral response.

## Results

Our experimental design consisted of six biological replicates of PBMCs, three of which were treated with Poly(I:C) as described further in Methods. RNA libraries were prepared from the six samples using the Lexogen QuantSeq kit or Illumina TruSeq kit (the latter will be referred to as RNA-Seq). In addition, RNA from the six samples were analysed with Clariom S Human microarrays. Following library generation with either QuantSeq or TruSeq kits and 75 bp single-end and paired-end sequencing, respectively, on the Illumina NextSeq500 the sequenced reads were mapped using two different strategies. First, they were mapped to the human genome using Tophat2^19^ with reads assigned to features using featureCounts of the Subread package^25^. For brevity, we refer to this pipeline as the Tophat method. As an alternative we performed quantification using Salmon^17^, which does not provide a base-by-base mapping, followed by summarisation to the gene level using the R package, Tximport^22^. We refer to this as the Salmon method. Our next-generation sequencing workflow produced four result sets (i) RNA-Seq mapped with Tophat, (ii) QuantSeq mapped with Tophat (iii) RNA-Seq quantified with Salmon (iv) QuantSeq quantified with Salmon. Our primary data sets consisted of approximately 92 M reads per sample for RNA-Seq and 30 M reads per sample for QuantSeq.

In addition to analysing our full data sets we also conducted analyses on a subset of our data, consisting of the typical number of RNA-Seq reads used in mammalian transcriptomics (30 M reads per sample) and Lexogen’s recommended coverage for QuantSeq (10 M reads per sample). Whilst our full data sets might be considered a gold standard, the subsets represent a more typical experimental design. It is appropriate to use less reads for QuantSeq as sequencing is only performed on fragments from the 3′ end of the transcript rather than the entire transcript. Herein, lies a major potential advantage of QuantSeq, in that less sequencing reduces the total cost of the experiment. However, our design also enables us to compare RNA-Seq and QuantSeq using the same number of reads in both sets (QuantSeq full data (30 M reads per sample) vs RNA-Seq subset (30 M reads per sample)). We use the following shorthand notation and colour scheme in the Figures throughout this manuscript (RNA-Seq with Tophat (“Rseq”, dark orange), QuantSeq with Tophat (“Qseq”, dark blue), RNA-Seq with Salmon (“RseqSal”, light orange), QuantSeq with Tophat (“QseqSal”, light blue), Microarray (green), where we have used a subset of the data we append “SS” to the name).

The Salmon quantification method avoids base-to-base mapping of the entire read and is therefore very fast. The computational time required for the Tophat mapping was around 60 times greater than for Salmon. On our computer cluster Salmon quantification took 10 mins per sample using 6 threads while Tophat took in the order of 10 hours using 6 threads. As Salmon does not perform base-to-base mapping of the entire read it does not produce bam files. The Tophat method produced bam files, each with a size of 6–10 GB. By contrast, Salmon produced an output file of feature abundance (sf file) for each sample which is in the order of 10 MB, that is, approximately 1/100 the size of a bam file.

### QuantSeq reads originate from the 3′ end and UTR of RNA transcripts

**Figure 1:**
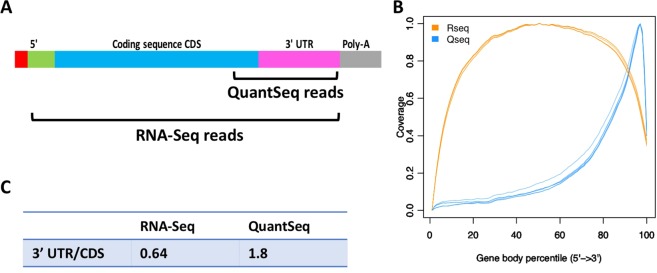
QuantSeq reads are predominantly from the 3′ end of RNA transcripts. (**A**) Schematic diagram of mRNA indicating the 3′ region from which QuantSeq reads are derived and noting that RNA-Seq reads may come from the entire transcript. (**B**) Coverage profile of our RNA-Seq samples (~92 M reads per sample) and QuantSeq samples (~30 M reads per sample) based on the number of reads covering each nucleotide position along the gene body including the 3′UTR and 5′UTR calculated using the geneBody_coverage.py of RSeQC. RNA-Seq samples 1–6, orange lines, QuantSeq samples 1–6, blue lines. (**C**) The ratio of reads aligned to 3′UTR versus CDS exons (Tags/Kb) calculated using the read_distribution.py script of RseQC.

We first analysed the location of reads generated by the QuantSeq and RNA-Seq methods. We expected the QuantSeq reads to align to the 3′UTR and the final exon/s of the coding sequence (CDS) whereas RNA-Seq reads cover the entire transcript (Fig. 1A). This was confirmed by calculating the average read coverage across genes using the python script geneBody_coverage.py from the RSeQC package^26^. The results showed that the six RNA-Seq libraries (orange lines in Fig. 1B) can be depicted by an inverted u-curve in which coverage plateaus in the middle of the transcript but is lower at both the 3′ and 5′ ends, whereas the QuantSeq reads peak at the 3′ end of the gene (blue lines in Fig. 1B). QuantSeq coverage is higher than RNA-Seq over the last 10% of the gene body including the 3′UTR. There is a great deal of variability in the length of the 3′UTR in human genes (range 60 bp – 4000 bp)^27^ and as QuantSeq produces a mean library fragment size between 335–456 bp (www.lexogen.com), reads will match both the coding sequence (CDS) and the 3′UTR. We expected that the ratio of 3′UTR reads to CDS reads in the QuantSeq data would be higher than in RNA-Seq, where the entire CDS is sampled. Comparing the reads per kilobase derived from the 3′UTR and the reads per kilobase of the CDS we did indeed find a three-fold increase in the 3′UTR/CDS ratio in QuantSeq compared to RNA-Seq (Fig. 1C).

We then compared the allocation of reads to genes in the RNA-Seq and QuantSeq data. We found that a higher percentage of the RNA-Seq reads were uniquely assigned to features compared with the QuantSeq reads (average 78% for RNA-Seq and 64% for Quant-Seq with TopHat; average 88% for RNA-Seq and 79% for QuantSeq with Salmon). The difference between RNA-Seq and QuantSeq was mainly due to the greater number of multimapped reads with QuantSeq (9% average for RNA-Seq versus 18% for QuantSeq). This indicates that a larger proportion of the QuantSeq single end 3′ reads can be aligned to multiple genomic regions. A decrease in uniquely mapped reads for QuantSeq has been proposed to arise from sequences found in 3′ regions that reduce the number of uniquely assignable reads^12^. Furthermore, our QuantSeq reads were single end reads whereas our RNA-Seq reads were paired end which will also affect the number of multimapped reads^28^. To summarise the mapping results, QuantSeq reads mapped towards the 3′ end of CDS and the 3′UTR where coverage was higher than for RNA-Seq but a higher percentage of reads were discarded compared to RNA-Seq.

Most eukaryotic genes harbour multiple polyadenylation sites (PAS) with most being located in the 3′UTR^29^. It is possible that the QuantSeq pipeline may fail to identify alternative polyadenylation isoforms thus affecting the overall gene count and expression level of these genes. To assess this we compared expression distributions for genes with 1, 2, 4, 6, 8 and 10 PAS. This analysis indicates that QuantSeq did not perform differently to RNA-Seq in quantifying genes with multiple PAS. Boxplots of these distributions are Supplementary Fig. S1.

### Protein coding and non-coding genes found by the different quantification methods

**Figure 2:**
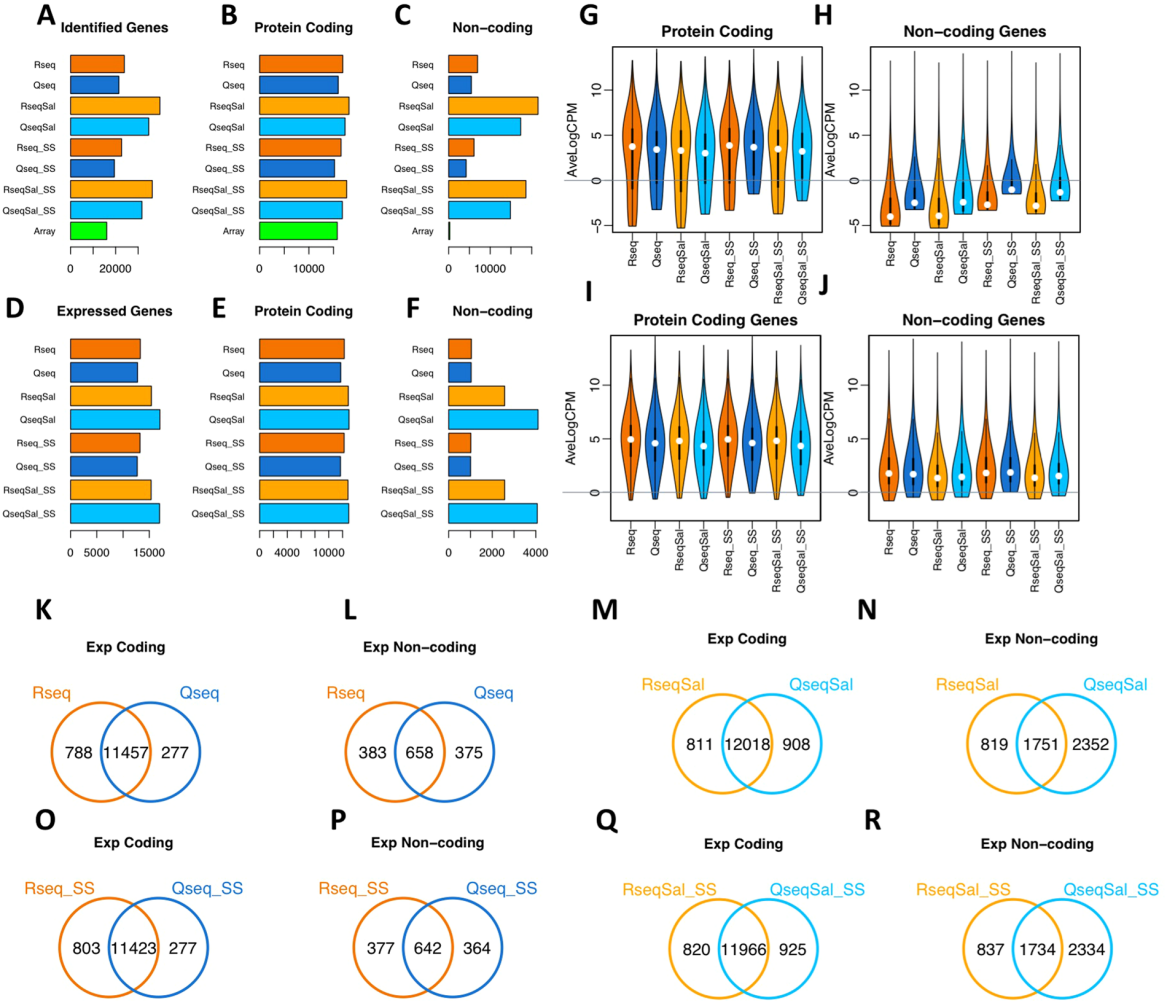
Comparing gene identification and gene expression for protein coding and non-coding genes.(**A**–**C**) Barplots comparing the total number of identified genes found in each of the datasets and splitting these into protein coding genes and non-coding genes. (**D**–**F**) Barplots comparing the total number of expressed genes found in each of the datasets and splitting these into protein coding genes and non-coding genes. (**G**–**H**) Violin plots showing distributions of average expression values (AveLogCPM) for protein coding genes and non-coding genes identified by the different methods. (**I**–**J**) Violin plots for all expressed genes (after filtering to retain only those genes with > 1 CPM in at least 3 samples, and with an associated gene symbol). (**K**–**R**) Venn diagrams showing pairwise comparison of expressed (‘Exp’) protein coding genes (**K**,**M**,**O**,**Q**) and non-coding genes (**L**,**N**,**P**,**R**). General colour scheme: RNA-Seq (Tophat): dark orange. QuantSeq (Tophat):dark blue, RNA-Seq (Salmon): light orange, QuantSeq (Salmon): light blue. Subsets (30 M reads per sample for RNA-Seq and 10 M reads per sample for QuantSeq are denoted with SS).

We next investigated the capacity of RNA-Seq and QuantSeq to detect protein coding and non-coding features (such as pseudogenes, antisense, snoRNA, lincRNA), together referred to as “genes”. A gene was “identified” if at least one read from one sample was mapped to that gene. The total number of identified genes are set out in Table 1 and compared by barplot in Fig. 2(A–C). Filtering of lowly-expressed genes was undertaken to arrive at a more robust list of genes we consider to be expressed. We used a typical filtering strategy to remove genes with low counts^30^, excluding genes with less than one count per million (1 CPM) in at least half of the samples. The remaining “expressed genes” were used in downstream differential expression analysis. The total number of expressed genes for each data set can be found in Table 1 and are plotted to provide a comparison between the methods (Fig. 2D–F). We found consistency between RNA-Seq and QuantSeq in the number of protein coding genes before filtering (Fig. 2B) and after filtering (Fig. 2E). There was more variability in the number of non-coding features (Fig. 2C,F). The most striking difference seen from the barplots is that more non-coding genes are included with Salmon quantification (Fig. 2C,F) and that this is more prominent for the QuantSeq data than the RNA-Seq data. Distribution plots (violin plots) of the expression levels of the protein coding and non-coding features showed a broad range of expression values, from very low to very high expression (Fig. 2G–J). The non-coding features were of generally low expression (Fig. 2H,J) and a significant proportion of these features were excluded after filtering (compare Fig. 2G–H,I–J). After filtering to remove lowly expressed genes we found consistent distributions of expression levels within the protein coding and non-coding data sets.

**Table 1:** Genes expressed in PBMCs as detected by RNA-Seq or QuantSeq and those that are differentially expressed after treatment with polyinosinic-polyctidylic acid.

	Identified Genes^a^	Protein Coding %^b^	Expressed Genes^c^	Protein Coding %^b^	DEGs^d^	Protein Coding %^b^
RNA-Seq (Tophat)^e^	23857	0.71	13286	0.92	1597	0.92
QuantSeq (Tophat)^f^	21425	0.75	12767	0.92	1129	0.92
RNA-Seq SS (Tophat)^g^	22664	0.73	13245	0.92	1247	0.94
QuantSeq SS (Tophat)^h^	19433	0.78	12706	0.92	958	0.93
RNA-Seq (Salmon)^e^	39657	0.46	15396	0.83	1795	0.85
QuantSeq (Salmon)^f^	34689	0.50	17026	0.76	1160	0.79
RNA-Seq SS (Salmon)^g^	36271	0.49	15354	0.83	1519	0.86
QuantSeq SS (Salmon)^h^	31676	0.53	16956	0.76	961	0.83
Microarray	16039	0.99			327	

^a^Number of genes with an associated gene symbol identified as having at least one read in at least one sample.

^b^The percentage of protein coding genes in the total number from the previous column.

^c^After filtering to remove those genes with <1 CPM in at least 3 samples.

^d^All differentially expressed genes with FDR < 0.05 and logFC > 1 determined as set out in Methods.

^e^All data being an average of 92 M reads per sample for RNA-Seq.

^f^All data being an average of 30 M reads per sample for QuantSeq.

^g^Subset (SS) of data being an average of 30 M reads per sample for the RNA-Seq dataset.

^h^Subset (SS) of data being an average of 10 M reads per sample for the QuantSeq dataset.

Clariom S arrays used for microarray analysis have probes for well-annotated genes rather than the full transcriptome of coding and non-coding features, with 99% of the detected genes being protein coding. The number of protein coding genes detected by microarray is similar to the number identified in our RNA-Seq and QuantSeq data sets (Fig. 2B). Overall, we see internal consistency in the number of protein coding genes across all the next-generation sequencing methods but variation in the number of non-coding genes with more found when using Salmon.

To compare the expression measures from RNA-Seq, QuantSeq and microarray, we calculated the Spearman’s rank correlation co-efficient of the average expression values (AveLogCPM) for all expressed genes (Table 2). The RNA-Seq and QuantSeq data was strongly correlated (Spearman’s rho 0.83, Tophat; 0.80, Salmon). We also found very high correlation when comparing the Tophat and Salmon mapping for either the RNA-Seq (Spearman’s rho 0.98) or QuantSeq (Spearman’s rho 0.93). Correlation between the RNA-Seq and microarray (Spearman’s rho 0.79) and QuantSeq and microarray (Spearman’s rho 0.7) was reduced but still revealed a high degree of correlation.

**Table 2:** Spearman’s Rank Correlation for Average expression of Expressed Genes.

	Rseq	Qseq	Rseq SS	Qseq SS	Rseq Sal	Qseq Sal	Rseq Sal SS	Qseq Sal SS	Array
Rseq	1	0.83	1	0.83	0.98	0.78	0.98	0.78	0.79
Qseq		1	0.83	1	0.82	0.93	0.82	0.93	0.7
Rseq SS			1	0.83	0.98	0.78	0.98	0.78	0.79
Qseq SS				1	0.82	0.93	0.82	0.93	0.7
RseqSal					1	0.8	1	0.8	0.79
QseqSal						1	0.8	1	0.68
RseqSal SS							1	0.8	0.79
QseqSal SS								1	0.68
Array									1

To compare the gene sets found by the different approaches, Venn diagrams were made for four pairwise comparisons of the coding and non-coding expressed genes (Fig. 2K–R). We saw a similar high overlap in the protein coding genes when comparing QuantSeq with RNA-Seq irrespective of quantification method, for both the full data set and the subsets (Tophat: Jaccard coef = 0.91, Salmon: Jaccard coef = 0.87). The non-coding genes accounted for a modest proportion of the full complement of expressed genes, 8% of the Tophat expressed genes and between 17–24% of the Salmon expressed genes. These subsets of non-coding genes show greater variability between RNA-Seq and QuantSeq (Tophat: Jaccard coef = 0.46, Salmon: Jaccard coef = 0.36). The most striking difference between the RNA-Seq and QuantSeq expressed genes can be seen in Venn diagrams comparing the non-coding genes found by Salmon (Fig. 2N,R). The QuantSeq expressed genes include 2352 non-coding genes not expressed in the RNA-Seq data. Overall, 72% of the QuantSeq exclusive genes, were non-coding RNA which represents a doubling or tripling in the proportion of non-coding RNA in the set of genes found to be exclusive to one method. Pairwise comparisons of the expressed protein coding and non-coding genes in each of our data sets can be found in Supplementary Table S1.

To summarise, with Tophat mapping the RNA-Seq data sets have a slightly higher number of expressed protein coding genes (~4% higher) than QuantSeq but a very similar number of non-coding genes (~1% higher). Furthermore, QuantSeq recovers around 94% of the protein coding genes and around 60–70% of the non-coding genes found by RNA-Seq. With Salmon quantification we found that RNA-Seq and QuantSeq produce a similar number of expressed protein coding genes (<0.1% difference) and again QuantSeq recovers around 94% of the protein coding genes found by RNA-Seq. However, we see a difference in the non-coding genes, with Salmon reporting a substantial increase in the number of non-coding genes from the QuantSeq data.

**Figure 3:**
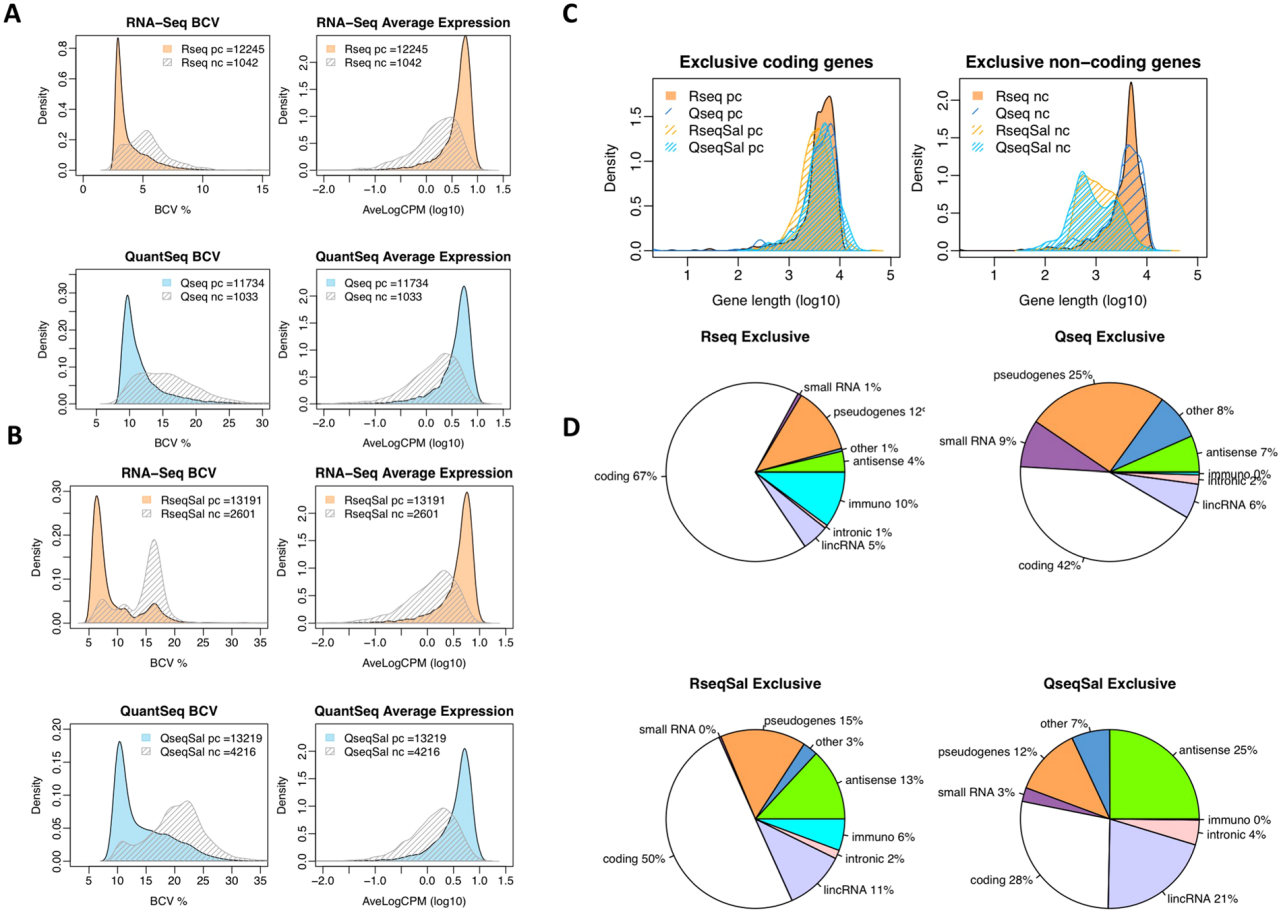
Comparing genes expressed in RNA-Seq or QuantSeq. Distribution plots of biological co-efficient of variation and average expression comparing the protein coding (pc) genes and non-protein coding (nc) genes for (**A**) Tophat data (**B**) Salmon quantification. (**C**) Distribution plots of gene length for genes expressed exclusively in RNA-Seq or QuantSeq. (**D**) Pie charts showing proportion of protein coding and non-coding gene types for the sets of genes expressed exclusively in RNA-Seq or QuantSeq, top row Tophat mapping, lower row Salmon quantification.

The substantial increase in the non-coding genes reported from Salmon analysis of the QuantSeq data raised the question of whether these observations are true. Characteristics of this data set may help to understand this issue. Firstly, the non-coding genes tend to be ‘noisy’ in that they have lower expression and higher variability between samples. This was evident when comparing average expression values and the biological co-efficient of variation (BCV) for the coding and non-coding genes (Fig. 3A,B). Secondly, we considered whether gene length could be a factor contributing to the differences seen. Distribution plots of gene length for the protein coding and non-coding genes expressed exclusively in QuantSeq or RNA-Seq were generated (Fig. 3C). We saw little difference in gene length distribution between QuantSeq and RNA-Seq in the protein coding genes. However, for the non-coding features we saw a marked shift in the distributions arising from Salmon quantification, with shorter non-coding features being seen in both the QuantSeq and RNA-Seq exclusive non-coding genes (Fig. 3C). Thirdly, we assessed the types of non-coding features which were being found exclusively in RNA-Seq or QuantSeq. Pie charts of the gene biotype are presented in Fig. 3(D). Interestingly, with RNA-Seq we saw an increase in the immunity related non-coding genes, the TR_V genes and the IG_V genes and with QuantSeq an increase in small RNAs, pseudogenes and antisense. In the Salmon-generated counts we saw an increase in lincRNA and antisense transcripts as compared to counts generated by Tophat. In summary the exclusive non-coding genes tended to be lowly expressed, variable across samples and of shorter length.

### Differential expression analysis

We next sought to understand the capacity of the QuantSeq, RNA-Seq and microarray methods to discover changes in gene expression between normal and Poly(I:C)-treated PBMCs. We performed differential expression analysis using (i) RNA-Seq with approximately 92 M reads/for each of the 6 samples (ii) QuantSeq with approximately 30 M reads/for each of the 6 samples (iii) RNA-Seq with approximately 30 M reads/for each of the 6 samples (iv) QuantSeq with approximately 10 M reads/for each of the 6 samples (v) Clariom S microarrays of the 6 samples. Each of the non-microarray experiments were quantified using the Tophat and also the Salmon methods. This produced 9 result sets in total. We used an FDR cut-off of <0.05 and incorporated a fold change threshold into the statistical testing using the TREAT approach^31^ to be more certain of obtaining biologically significant results.

**Figure 4:**
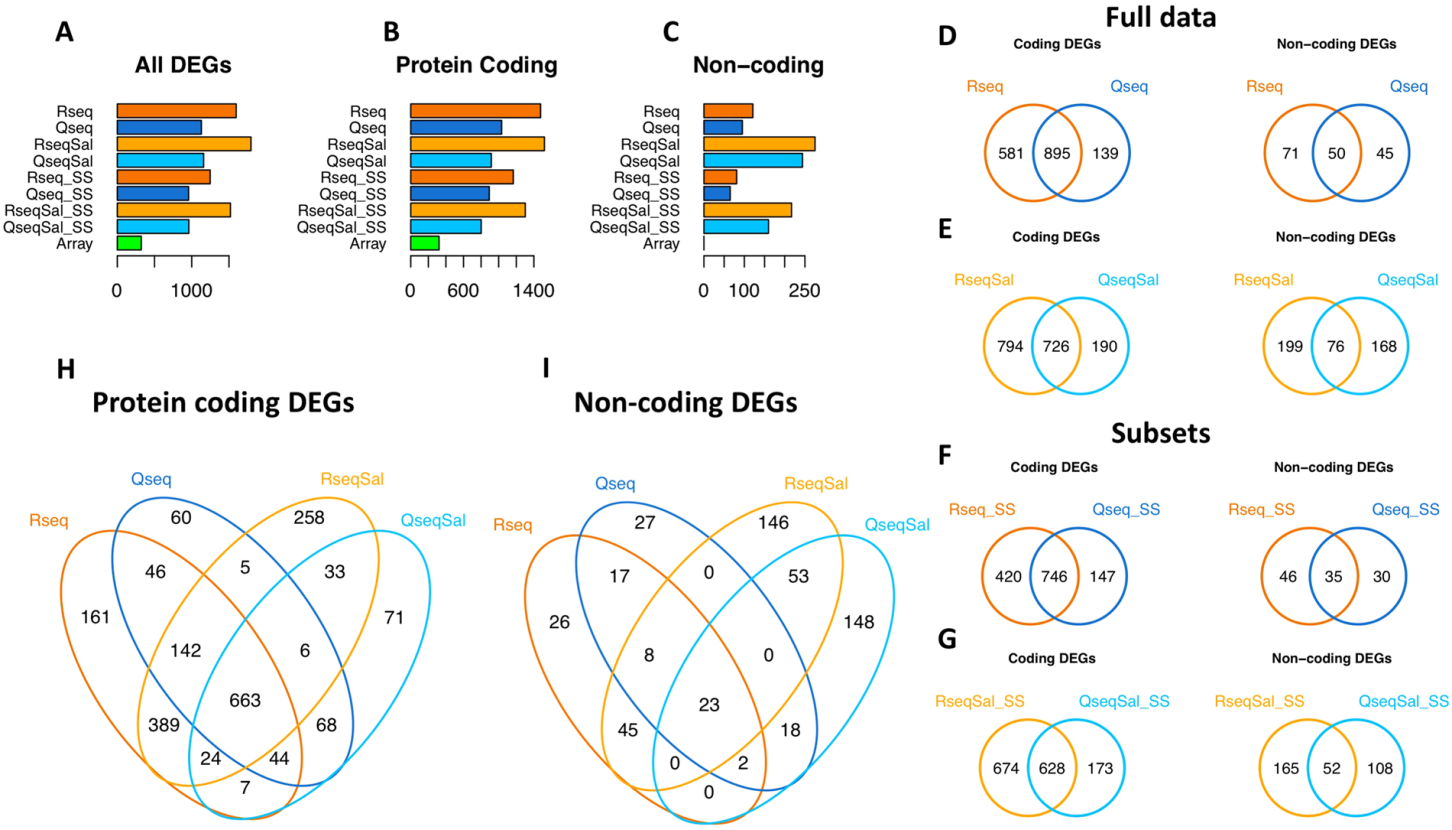
Comparing differentially expressed genes (DEGs). (**A**–**C**) Barplots comparing the total number of differentially expressed genes (DEGs) found in each of the result sets (**A**) and split into the number of protein coding DEGs (**B**) and the number of non-coding DEGs (**C**). (**D**–**G**) Venn diagrams comparing RNA-Seq and QuantSeq DEGs, split into protein coding genes (left side) and non-coding genes (right side). (**H**–**I**) Venn diagrams of DEGs found with RNA-Seq and QuantSeq using Tophat or Salmon quantification. General colour scheme: RNA-Seq (Tophat): dark orange. QuantSeq (Tophat):dark blue, RNA-Seq (Salmon): light orange, QuantSeq (Salmon): light blue, Microarray: green. Subsets (30 M reads per sample for RNA-Seq and 10 M reads per sample for QuantSeq are denoted with SS).

The total number of differentially expressed genes (DEGs) found in each different result set are given in Table 1 and are compared in barplots (Fig. 4A–C). RNA-Seq identified more DEGs than QuantSeq (~30–40% with Tophat and ~50–60% with Salmon, Fig. 4A). Microarray identified far fewer DEGs, around 75–80% less than those found by RNA-Seq or QuantSeq with only 2% not being found by the other methods. If the DEGs were split into protein coding and non-coding genes (Fig. 4B,C respectively), it is apparent that RNA-Seq identifies a greater number of both protein coding and non-coding DEGs than QuantSeq. Figure 4C also demonstrates that Salmon quantification led to an increase in the number of non-coding DEGs compared to Tophat mapping.

We carried out a pairwise comparison of all RNA-Seq and QuantSeq DEGs and calculated the number of protein coding and non-coding DEGs shared in each pairwise comparison (Supplementary Tables S2). Venn diagrams for four pairwise comparisons of both protein coding and non-coding DEGs are shown in Fig. 4(D–G). These comparisons showed that around 80% of the QuantSeq protein coding DEGs are also identified by RNA-Seq. There was less agreement between the RNA-Seq and QuantSeq methods for the non-coding DEGs. However, these subsets of non-coding genes form a smaller percentage of the entire set, less than 8% of the DEGs produced using Tophat are non-coding whereas up to 21% are non-coding if Salmon quantification is used. We also found that the subsets comprised of 30 M and 10 M reads per sample for RNA-Seq and QuantSeq respectively versus the full data set of 92 M reads per sample for RNA-Seq and 30 M reads per sample for QuantSeq recovered 80–85% of the DEGs found by the full data sets (Table 1, Fig. 4A, Supplementary Table S2). Furthermore, the RNA-Seq subset of 30 M reads per sample identified around 10% more DEGs than the full QuantSeq dataset, also having 30 M reads per sample. Comparisons of the DEGs found in RNA-Seq and QuantSeq with both Tophat and Salmon quantification are presented in Venn diagrams (Fig. 4(H–I)). Interestingly, this shows that a core set of coding genes are found as differentially expressed, irrespective of approach, but that the non-coding genes found are strongly influenced by the sequencing and read mapping approach taken.

### Why are some genes differentially expressed by RNA-Seq or QuantSeq but not by both?

**Figure 5:**
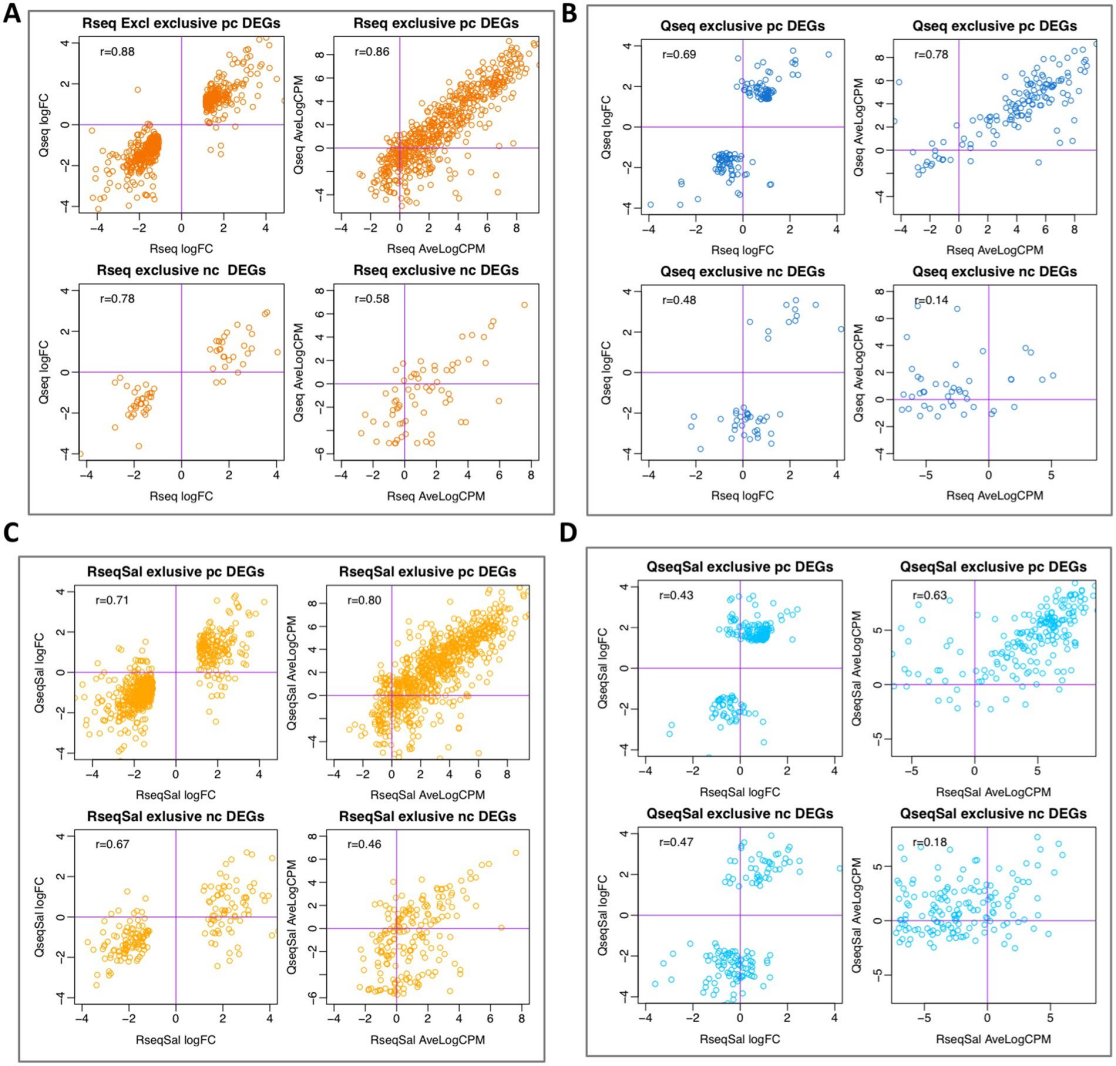
Comparing fold change and expression in DEGs found exclusively by RNA-Seq or QuantSeq. Scatterplots of fold change and average expression comparing values derived from RNA-Seq and QuantSeq data for the DEGs found by one method and not the other. Each panel, top row LogFC and AveLogCPM for protein-coding (pc) DEGs, bottom row non-coding (nc) DEGs. (**A**) DEGs found by RNA-Seq not QuantSeq using Tophat. (**B**) DEGs found by QuantSeq not RNA-Seq using Tophat. (**C**–**D**) using Salmon.

**Figure 6:**
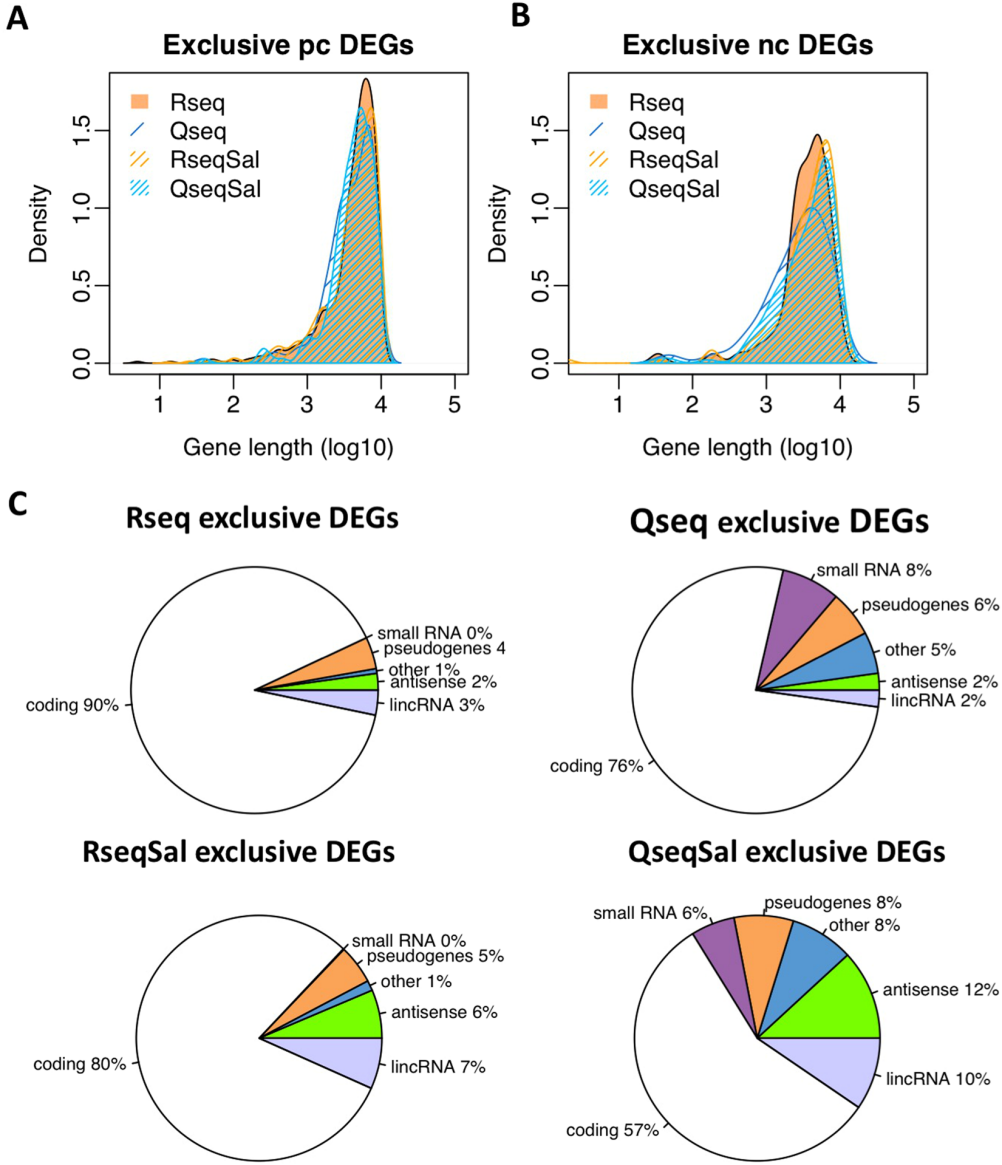
Gene length and biotype in the exclusive DEGs. (**A**,**B**) Distribution plots of gene length for the non-common differentially expressed protein coding (pc) genes and non-coding (nc) genes (DEGs) show a slight shift towards genes with a shorter sequencing region in the QuantSeq data, most noticeable in the non-coding genes. (**C**) Classification of these DEGs by biotype reveals enrichment of non-coding RNAs, particularly in the QuantSeq data.

We examined the DEGs found in RNA-Seq but not in QuantSeq and vice versa to identify any characteristics which could help to explain why these genes are identified as being differentially expressed by one method but not the other. We calculated Spearman’s correlation coefficient for the logFC values and average expression values of protein coding DEGs found by RNA-Seq but not QuantSeq. This revealed a high correlation of the fold change and average expression (logFC r = 0.88, AveExpr r = 0.86 (Tophat, n = 576)). When we did the same for the 780 protein coding DEGs found exclusively in RNA-Seq using Salmon we found reduced but still strong correlation (logFC r = 0.71, AveExpr r = 0.80 (Salmon, n = 780)). This demonstrates that the expression profile of DE protein coding genes found uniquely by RNA-Seq is likely due to the failure of the QuantSeq data to meet the thresholds for differential expression. There were far fewer DEGs found exclusively in the QuantSeq data. In this case we found reduced correlation of fold change and average expression (logFC r = 0.69, AveExpr r = 0.78 (Tophat, n = 139) and (logFC r = 0.43, AveExpr r = 0.63 (Salmon, n = 196)). Scatterplots of fold change and average expression for genes found to be differentially expressed exclusively in RNA-Seq or QuantSeq are presented in Fig. 5. We found lower correlation for the non-coding DEGs, however there were also fewer of these genes. Looking more closely at the DEGs unique to QuantSeq, we found a slight shift towards shorter non-coding genes (Fig. 6B) but similar length distributions for the protein coding genes (Fig. 6A). We also saw that a greater proportion of the QuantSeq exclusive DEGs were non-coding genes (Fig. 6C), with a notable increase in the small RNAs. The small RNAs (snoRNA, scRNA) are by definition short sequences and accordingly the two factors, gene length and biotype are related.

### Biological insight and gene set analysis

**Figure 7:**
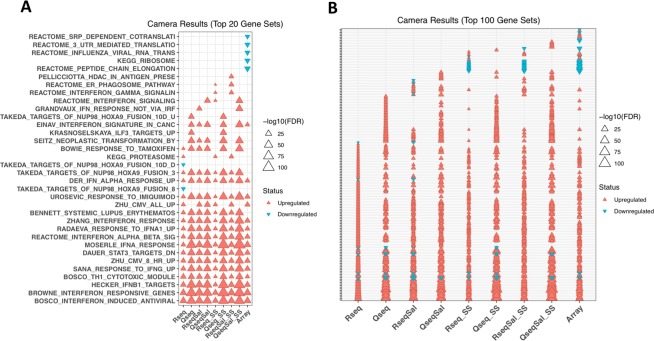
Gene Set analysis identifies antiviral signatures. Gene set analysis using Camera identifies enrichment of gene sets involving antiviral and inflammatory responses. (**A**) The top 20 gene sets arising in each of the data sets are plotted, as the top 20 are not exactly the same for each data set the total number of gene sets is greater than 20 in this plot. (**B**) The top 100 gene sets are plotted as the top 100 are not exactly the same for each data set the total number of gene sets is greater than 100 in this plot. Columns are ordered as RNA-Seq (Tophat), QuantSeq(Tophat), RNA-Seq (Salmon), QuantSeq (Salmon), RNA-Seq subset (30 M) (Tophat), QuantSeq subset (10 M) (Tophat), RNA-Seq subset (30 M) (Salmon),QuantSeq subset (10 M) (Salmon).

The endpoint of these analyses is to obtain insight into the biological or functional differences between the conditions being compared. We performed gene set analysis using the Camera function^32^ to obtain sets of functionally related genes associated with the poly(I:C) treatment. Gene Set Analysis is based on the principle of co-regulation; that genes which are functionally related are likely to have similar expression patterns. This analysis method considers the expression values of all expressed genes rather than being restricted to a group of DEGs chosen by an arbitrary cut-off (such as, FDR < 0.05 and LogFC > 1). In the top 20 gene sets identified in each of our data sets (Fig. 7A) we saw a strong signature of up-regulation and that the most significant gene sets are those contributing to the interferon induced anti-viral response, including a gene set previously described by us, the Bosco Interferon Induced Antiviral Module^33^. These highly significant gene sets were detected by all versions of the RNA-Seq and QuantSeq methods tested, including the subset data. Furthermore, we saw agreement in the microarray data set in these gene signatures. The microarray gene sets included 5 down-regulated sets, these were also significantly down-regulated in the RNA-Seq and QuantSeq data under both quantification methods but did not fall within the top 20. These findings are consistent with our expectations for this model system and serves as proof of principle that each of the expression quantification methods is detecting differential expression in the groups of genes we expect for this system. When we looked more broadly at the top 100 gene sets we still saw good agreement across the methods (Fig. 7B). 95 of the top 100 gene sets found by QuantSeq were also found to be significantly different in the RNA-Seq data and all of the top 100 gene sets identified by RNA-Seq were found to be significant in the QuantSeq data. Barcode plots which show the fold changes for the genes in the Bosco Interferon Induced Antiviral Module can be found in Supplementary Fig. S3.

Gene set analysis is particularly powerful as it avoids the problem of some genes failing to meet an imposed FDR cut-off in order to be classified as differentially expressed. However, the use of a restricted set of DEGs in functional analysis is also a common analytical approach. If we took only the DEGs and compare the top gene ontology terms and top KEGG pathways enriched in those lists we still found good agreement between RNA-Seq and QuantSeq with approximately 90% of the top 500 gene ontology functional classifications common to RNA-Seq and QuantSeq and 70% of the top 20 KEGG pathways (Supplementary Fig. S4). The four top KEGG pathways that appeared in each of our four data sets are Cytokine-cytokine receptor interaction, NOD-like receptor signalling pathway, Herpes simplex infection and Influenza A.

**Figure 8:**
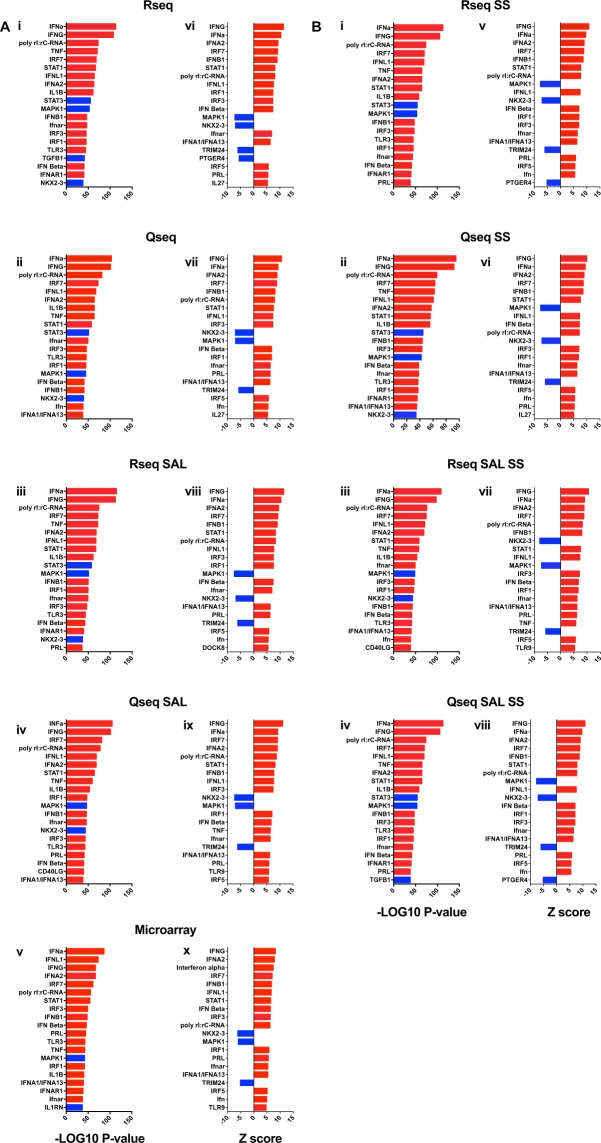
Identification of Transcriptional Regulators (TRs) of the polyI:C response in PBMCs. Molecular drivers of gene expression changes were inferred using Upstream Regulator Analysis on the full expression sets (**A**), and the subset data (**B**). Drivers are ranked by P-value (**A** (i-v) and **B** (i-iv)) and activation Z score (**A** (vi-x) and **B** (v–viii)). Red bars indicate pathway activation (activation Z-score > 2.0) and blue bars indicate pathway inhibition (activation Z-score < −2.0).

Finally, we performed Upstream Regulator Analysis (Ingenuity Systems) on the DEGs from all four datasets and the microarray dataset (Fig. 8A), and the four subset data sets (Fig. 8B). This analysis uses prior knowledge to identify transcriptional regulators (TR) that drive the observed gene expression changes to gain further biological insight. Two statistical measures are presented; (1) the overlap p-value, which measures the overlap between the DEGs and the target genes that are regulated by a TR (Fig. 8A (i–v),B (i–iv)); and (2) the activation Z-score, which measures the pattern match between expected relationship direction of a TR (activation or inhibition) and observed gene expression changes (up/downregulation) (Fig. 8A(vi-x),B(v-viii)). As illustrated in Fig. 8, PolyI:C is a top driver of gene expression changes in all datasets and the microarray data. Furthermore, the top drivers in all 5 datasets are dominated by well-known antiviral (type I and III interferons, IFNG, IRF3 and IRF7) and proinflammatory genes (TNF and IL1b). When the data sets are subset (Fig. 8B) the same key response drivers emerge as seen for the full data sets.

## Discussion

In this study we compared QuantSeq to RNA-Seq and microarrays and assessed its ability to return similar gene expression profiles and identify differentially expressed genes. This was done in a test case involving PBMCs stimulated with a viral double-stranded RNA mimic. As well as evaluating QuantSeq we also compared a new quantification method, Salmon, with Tophat2. Our design allowed us to compare the effect of library preparation (QuantSeq vs RNA-Seq), quantification method (Tophat2 vs Salmon) and sequencing depth (Full datasets vs subsets), on the identification of transcribed genes and the calling of DEGs.

Overall, we saw consistency in the expressed protein coding genes, with QuantSeq finding 94% of the expressed protein coding genes found by RNA-Seq, irrespective of quantification method. Comparing QuantSeq and RNA-Seq to microarray analysis we also found high correlation between ranked expression levels for the protein coding genes. We found reduced correlation and more variability in the non-coding genes which comprised between 8–24% of the expressed genes. At the outset it should be borne in mind that our library preparation depended on the presence of a polyA tail on the transcripts to be sequenced. As non-coding RNA transcripts fall into three categories, (i) those with a polyA tail, (ii) those without a poly A tail and (iii) those which are bimorphic (appearing both with and without a polyA tail)^34^ we would not expect to see the full population of non-coding RNA in our data sets. Using Tophat mapping we saw a similar number of non-coding features in the RNA-Seq and QuantSeq experiments (~1000). However, when we employed Salmon quantification we found an overall increase in non-coding features with a greater number being identified in the QuantSeq experiment, QuantSeq (~ 4000) versus RNA-Seq (~2500).

We also found a marked shift in the length distribution of the non-coding features with Salmon identifying a population of short non-coding transcripts, including a number of snRNA and snoRNA with a length less than 300 bp (around 10% of the Salmon/QuantSeq exclusive non-coding features). The remaining non-coding genes were enriched for antisense and lincRNA. It has previously been reported that most lincRNA are polyadenylated^35^ which is consistent with our sequencing approach. Interestingly, it has been reported that lincRNAs have similar sequence and structural features to 3′UTR^36^.

In summary, we found high concordance in the expressed protein coding genes across the methods tested. The small proportion of discordant genes tended to be non-coding genes with lower expression and shorter length than the concordant genes. Other benchmarking studies contrasting RNA-Seq quantification methods have also reported that non-concordant genes tend to have lower expression and shorter length^28,37,38^.

Performing differential expression analysis to assess the effect of Poly(I:C) on gene expression we found that that library preparation method (QuantSeq vs RNA-Seq) had a greater effect on our differential expression results than quantification method or read depth. RNA-Seq identified a greater number of DEGs than QuantSeq irrespective of quantification method. Nevertheless, a high percentage of the protein coding DEGs found by QuantSeq were also common to RNA-Seq (89% using Tophat, 79% using Salmon). Again, there were fewer differentially expressed non-coding DEGs and more variability in the sets found by QuantSeq and RNA-Seq. When we looked more closely at the DEGs called by RNA-Seq but not by QuantSeq we found that a majority had similar expression characteristics (logFC and average expression) whilst failing to meet our logFC and FDR cut-offs to be classified as DEGs. Both QuantSeq and RNA-Seq clearly outperformed microarray in identifying differentially expressed genes.

We found remarkable consistency in the gene sets emerging as most biologically significant in our differential expression analysis. The most significant gene set in each of our data sets was the Bosco Interferon Induced Anitviral Module. This gene signature meets our expectation for our test case in which we anticipated elevation of gene sets involved in inflammation and anti-viral responses. We see this result clearly emerging from all the methods tested including when we use subsets of data consisting of 10 M QuantSeq reads per sample. In addition, we see similar transcription factors acting as drivers of the changed gene expression in our data sets. We are mindful however that not all biological investigations will be as clear cut as our test case, which involved a strong biological response. It may be that we would see a divergence of RNA-Seq and QuantSeq outcomes in a more nuanced scenario.

As QuantSeq only sequences the 3′ end of the RNA fragment less reads should be required than for RNA-Seq where reads are derived from the entire fragment. Lexogen suggests that 10 M reads per sample are adequate for quantification. We tested this and compared results using 10 M reads per sample to those obtained with three times that read depth. We found very little difference in the number of expressed genes (less than 0.5%) using the QuantSeq subset compared to the full data set. When we performed differential expression analysis we found a modest decrease in the number of DEGs of around 15%. Nevertheless, the same biological signals were highlighted in the subset data as found for the full data set. Comparing QuantSeq with 30 M reads per sample to RNA-Seq with 30 M reads per sample we found that RNA-Seq had a small increase in expressed genes and around a 10% increase in DEGs compared to QuantSeq. This draws us to conclude that QuantSeq data consisting of 10 M reads per sample is adequate for straight forward gene expression analysis and that little is to be gained by deeper sequencing in this case.

As well as evaluating QuantSeq and RNA-Seq we have compared the new quantification method, Salmon with Tophat2. Quantification with Salmon took less than 10 minutes per sample (6 CPUs) delivering the speed advantages we expected^17^. The resulting data file was around 1/100 the size of a bam file. The speed of processing and reduced storage requirements are important considerations in applying these techniques to projects involving many samples such as longitudinal cohort studies, single cell RNA-Seq and clinical applications.

## Conclusions

Overall, this study shows that combining QuantSeq with a fast quantification method such as Salmon is capable of providing a clear diagnostic signal in a relatively quick and inexpensive manner and may therefore be suited to high throughput clinical applications or to single cell transcriptomics. An additional benefit of QuantSeq is that the high read coverage over the 3′UTR is likely to be of particular interest to those studying this important gene regulatory region. It should be noted however that the QuantSeq - Salmon workflow is restricted to assessing gene expression changes and does not provide information regarding mutations/base changes or novel transcripts. This pipeline is ideally suited to situations where straight forward gene expression changes would assist in diagnostics or treatment.

## Methods

### Cellular stimulation and RNA preparation

Whole blood was obtained from a healthy adult volunteer, and peripheral blood mononuclear cells (PBMC) were isolated over Ficoll and cryobanked as previously described^39^. The cryobanked PBMC were thawed and cultured in AIM-V medium (Gibco) with 50 μg/ml polyI:C extract (Invivogen), or medium alone (control) in triplicate. After 24 h, cells were harvested and total RNA was extracted using TRIzol (Invitrogen) followed by purification on an RNeasy column (Qiagen)^40^. Quality of the total RNA was assessed on the Bioanalyser (Thermofisher) and RIN scores were ≥8 for all samples. Six samples (3 treated with Poly(I:C) and 3 controls) were used in each of the RNA-Seq, QuantSeq and microarray experiments.

### RNA library preparation

RNA-Seq libraries were prepared using the Illumina TruSeq Stranded mRNA Prep Kit according to the manufacturer’s instructions. The RNA-Seq libraries were sequenced using the Illumina NextSeq 500 to produce 75 bp paired-end reads for each sample. The QuantSeq libraries were prepare using Lexogen’s QuantSeq 3′ mRNA-Seq Library Prep Kit for Illumina, according to the manufacturer’s instructions. The QuantSeq libraries were sequenced using the Illumina NextSeq 500 to produce 75 bp single-end reads for each sample.

Library preparation and sequencing was done at the Ramaciotti Centre for Genomics (University of New South Wales, Australia).

### Microarray

Clariom S Human Assays (ThermoFisher) were used for microarray analysis. The analysis was undertaken in accordance with the manufacturer’s instructions, by the Ramaciotti Centre for Genomics.

### Bioinformatic analysis of RNA-Seq and QuantSeq reads

The QuantSeq reads were trimmed to remove the first 12 bp from the 3′ end in accordance with Lexogen’s recommendation. For this we used trim_galore (v 0.4.4) with the command–three_prime_clip_R1 12. RNA-Seq reads were not trimmed.

The RNA-Seq and QuantSeq reads (fastq files) were mapped to the Ensembl *Homo sapiens* genome (GRCh38). Mapping was performed with Tophat2 (v 2.0.12)^41^ calling Bowtie2 (v 2.2.3)^19^. The featureCounts function of Subread (v 1.4.6-p5)^25^ was used to generate counts of reads uniquely mapped to annotated genes using the GRCh38 gtf file. In addition we used the abundance quantification method Salmon^17^ to calculate transcript abundance based on the transcript fasta files downloaded from ensemble-release87, (Homo_sapiens.GRCh38.cdna.all.fa and Homo_sapiens.GRCh38.ncrna.all.fa). We ran Salmon with the following default settings for the RNA-seq data: quant -i transcripts_index -l ISR -1 *R1*fastq* -2 *R2*fastq* -o $output -p 6–posBias–gcBias–seqBias –writeUnmappedNames. For the QuantSeq data which would only capture the 3′ end of the transcript we changed the settings to: quant -i transcripts_index -l SR -r *R1_trimmed.fq* -o $output -p 6 –writeUnmappedNames. The Bioconductor package tximport^22^ was used to convert transcripts to gene counts which could be used in downstream differential expression analysis.

We used the polyA_DB v3 database^29^ to ascertain polyadenylation sites (PAS). We selected those sites which are found in the 3 ‘UTR and which have an extension. This yielded 32969 PASs found in 9967 genes. We took those genes which have 1, 2, 4, 6, 8 and 10 PASs and compared expression (AveLogCPM) in RNA-Seq and Quant-Seq by plotting boxplots of distribution.

Differential expression analysis was performed using functions from the Bioconductor packages edgeR (v 3.14.0)^42^ and voom limma (v 3.32.7)^43,44^. We excluded lowly expressed genes and tested those genes with expression of at least 1 CPM (counts per million) in at least three samples. Counts were normalized using the TMM method and generalized linear models were used for differential expression analysis, using the functions glmQLFit and glmTreat^31^ with lfc = log2(2). In all cases differentially expressed genes were defined as those genes with a Benjamini-Hochberg corrected p value less than 0.05^45^.

### Bioinformatic analysis of microarrays

The Bioconductor packages oligo and limma were used to perform normalization and differential expression analysis. The probes were annotated using the database clariomshumantranscriptcluster.db_8.6.0, available through Bioconductor. Normalization was performed using the Robust Multichip Average (RMA) algorithm. Linear models were constructed for all samples and the contrast of interest in this case the PBMCs with and without Poly(I:C) treatment was tested using empirical Bayes moderated t tests. The Treat function was used with lfc = log2(2). In all cases differentially expressed genes were defined as those genes with a Benjamini-Hochberg corrected p value less than 0.05.

### Gene set analysis

Gene set analysis was performed using the camera function^32^ within limma. The genes tested for differential expression were evaluated against the human_c2_v5p2 gene set collection downloaded from http://bioinf.wehi.edu.au. The camera algorithm analyses enrichment of genes in a set of interest and produces p-values and FDR values for the gene set being up-regulated or down-regulated in the data.

### Gene ontology and KEGG pathway enrichment and upstream regulator analysis

The most highly significant enriched gene ontology (GO) terms were assessed using the fea_topGO function of the package FGNet package^46^. The most significant KEGG pathways were assessed using the kegga function of limma. The input for these functions were the lists of DEGs derived in our four data sets. Molecular drivers of the gene expression responses were identified utilizing upstream regulator analysis in the Ingenuity Pathway Analysis package (Qiagen Bioinformatics)^47^.

### Ethics approval and consent to participate

This study was performed in accordance with the National Statement on Ethical Conduct in Human Research from the National Health and Medical Research Council. The study was approved by the Human Research Ethics Committee at the University of Western Australia. Written informed consent was obtained from study participants.

## Supplementary information

Supplementary Information

## Data Availability

The RNA-Seq sequencing data has been submitted to the NCBI Gene Expression Omnibus (GEO) (https://www.ncbi.nlm.nih.gov/geo/) and under accession number GSE123523.
