# Supplementary material for: QuantSeq. 3′ Sequencing combined with Salmon provides a fast, reliable approach for high throughput RNA expression analysis
Source: Sci Rep. 2019 Dec 11;9:18895. doi: 10.1038/s41598-019-55434-x (PMC6906367; doi:10.1038/s41598-019-55434-x)
Supplement: Supplementary file 1 — Supplementary Information [file 41598_2019_55434_MOESM1_ESM.pdf]

Supplementary Information

QuantSeq 3' Sequencing combined with Salmon provides a fast, reliable approach for high throughput RNA expression analysis

Susan M. Corley[1*], Niamh M. Troy[2], Anthony Bosco[2], Marc R.Wilkins[1]

1. Systems Biology Initiative, School of Biotechnology and Biomolecular Sciences, UNSW Sydney, New South Wales, Australia
2. Telethon Kids Institute Australia, The University of Western Australia, Perth, Australia

Supplementary Figure S1

Boxplots showing distribution of expression values for genes having 1, 2, 4, 6, 8 or 10 polyadenylation sites (PASs). General colour scheme: RNA-Seq (Tophat): dark orange. QuantSeq (Tophat):dark blue, RNA-Seq (Salmon): light orange, QuantSeq (Salmon): light blue, Microarray: green.

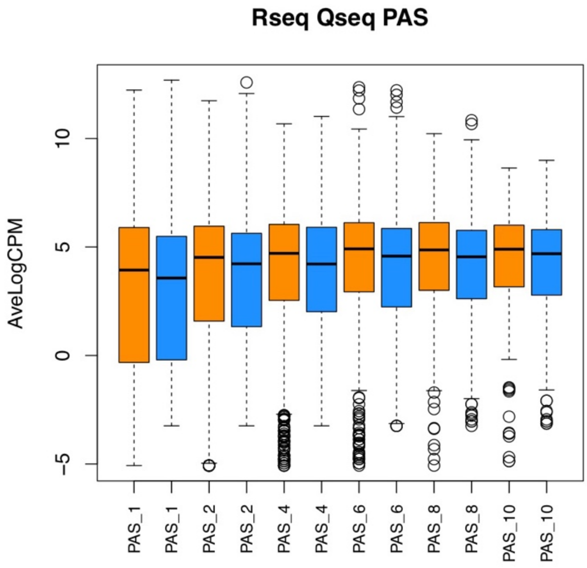

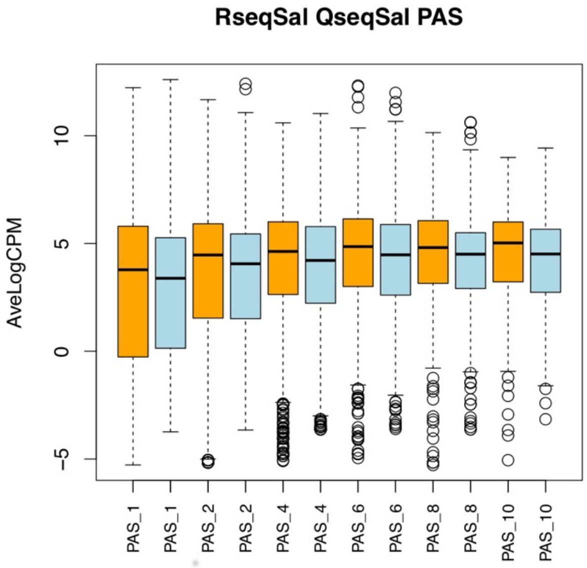

Supplementary Figure S2

Venn diagrams showing overlap in the differentially expressed genes in the Poly(I:C) treated samples derived using 2 different methods of library preparation (RNA-Seq or QuantSeq), 2 different methods of quantification (Tophat2 or Salmon) and different numbers of reads (full datasets comprising ~ 90 M reads per sample (RNA-Seq) and 30 M reads per sample (QuantSeq) versus subsets of 30 M reads per sample (RNA-Seq) and 10 M reads per sample (QuantSeq). The Jaccard similarity co-efficient appears under each Venn diagram. Row 1: comparison based on sequencing depth, Row 2: comparisons based on quantification method and Row 3: comparisons based on library preparation method. General colour scheme: RNA-Seq (Tophat): dark orange. QuantSeq (Tophat):dark blue, RNA-Seq (Salmon): light orange, QuantSeq (Salmon): light blue, Microarray: green.

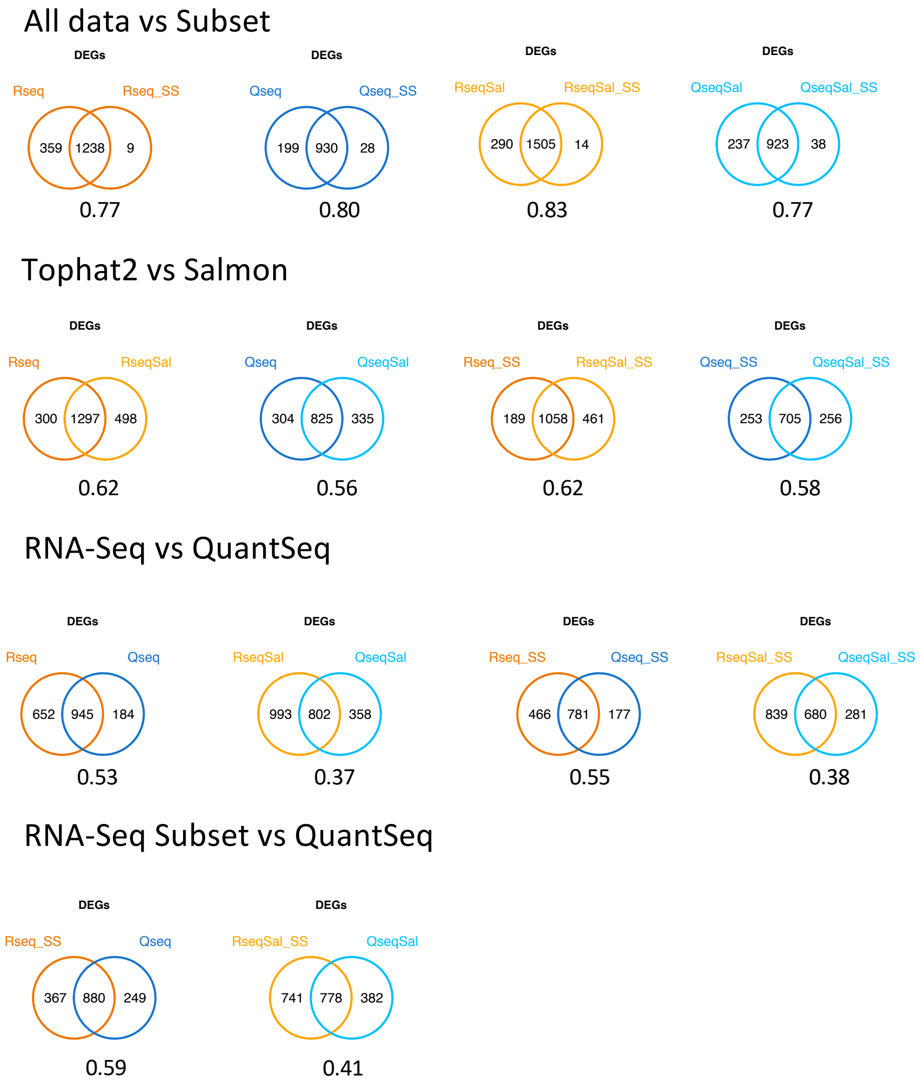

Supplementary Figure S3

Barcode plots showing the genes in the Bosco Interferon Induced Antiviral module in each of the four data seta. Each bar on the plot represents a gene and it's logFC is on the y-axis.

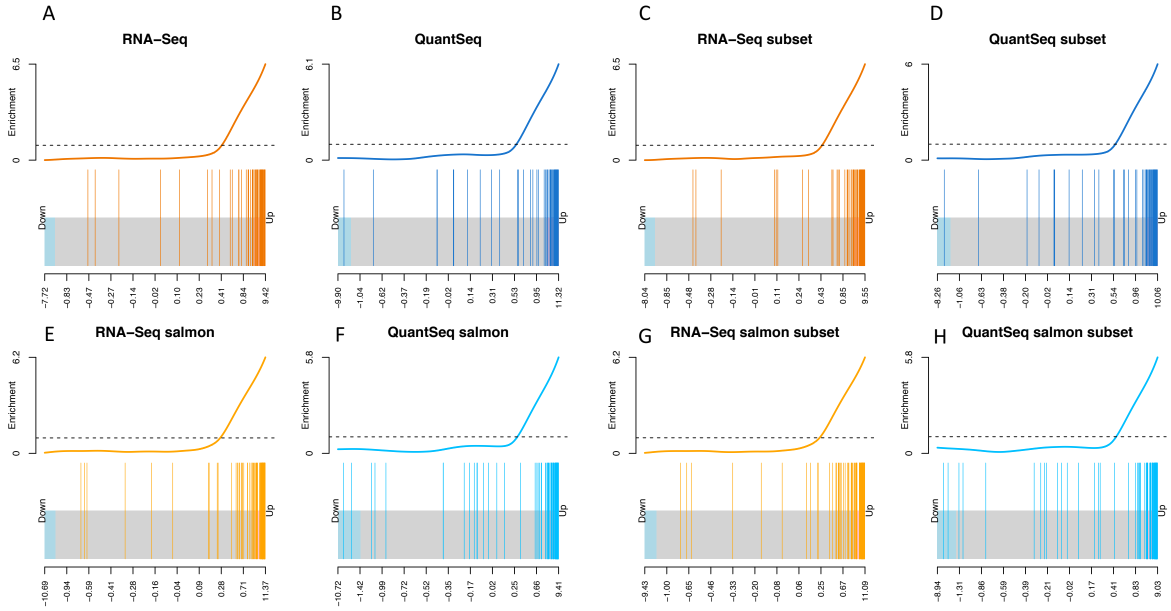

Supplementary Figure S4

Venn diagrams of overlap in enriched GO terms and KEGG pathways derived from the differentially expressed genes found in each data set.

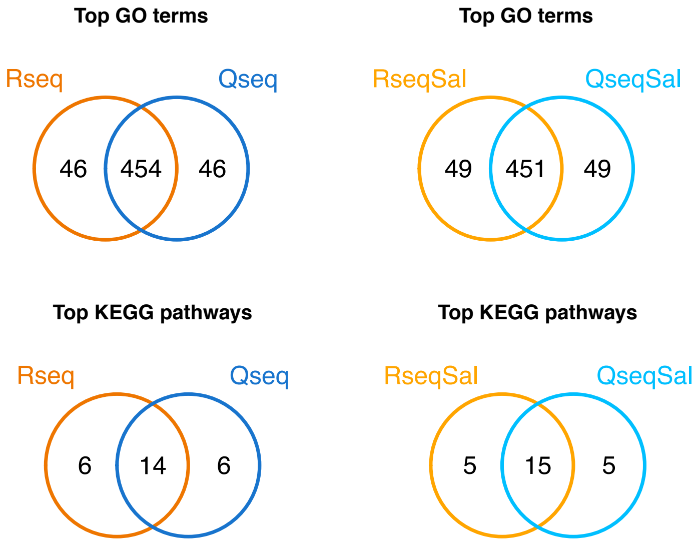

1. Cytokine-cytokine receptor interaction
2. NOD-like receptor signaling pathway
3. Herpes simplex infection
4. Influenza A

Supplementary Table S1

Pairwise comparisons of the expressed genes (protein coding and non-coding genes) in each of our data sets.

Coding	Rseq (12245)	Qseq (11734)	RseqSal (12829)	QseqSal (17026)	Rseq_SS (12226)	Qseq_SS (12706)	RseqSal_SS (12786)
**Qseq (11734)**	11457						
**RseqSal (12829)**	11720	11078					
**QseqSal (17026)**	11182	11085	12018				
**Rseq_SS (12226)**	12176	11450	11695	11175			
**Qseq_SS (12706)**	11425	11610	11053	11044	11423		
**RseqSal_SS (12786)**	11681	11059	12734	11997	11665	11030	
**QseqSal_SS (12891)**	11154	11059	11984	12807	11148	11024	11966

Non-coding	Rseq (1041)	Qseq (1033)	RseqSal (2570)	QseqSal (4103)	Rseq_SS (1019)	Qseq_SS (1006)	RseqSal_SS (2571)
**Qseq (1033)**	658						
**RseqSal (2570)**	717	504					
**QseqSal (4103)**	538	702	1751				
**Rseq_SS (1019)**	1002	657	707	530			
**Qseq_SS (1006)**	643	974	494	681	642		
**RseqSal_SS (2571)**	724	509	2490	1753	714	497	
**QseqSal_SS (4068)**	536	694	1737	3922	530	675	1734

Supplementary Table S2

Pairwise comparisons of the differentially expressed genes (protein coding and non-coding genes) in each of our data sets.

Coding	Rseq (1476)	Qseq (1034)	RseqSal (1520)	QseqSal (916)	Rseq_SS (1166)	Qseq_SS (893)	RseqSal_SS (1302)
Qseq (1034)	895						
RseqSal (1520)	1218	816					
QseqSal (916)	738	781	726				
Rseq_SS (1166)	1160	836	1049	697			
Qseq_SS (893)	784	869	724	724	746		
RseqSal_SS (1302)	1116	783	1294	708	1003	703	
QseqSal_SS (801)	654	698	643	774	624	674	628
Non-codiing	Rseq (121)	Qseq (95)	RseqSal (275)	QseqSal (244)	Rseq_SS (81)	Qseq_SS (65)	RseqSal_SS (217)
Qseq (95)	50						
RseqSal (275)	76	31					
QseqSal (244)	25	43	76				
Rseq_SS (81)	78	44	56	24			
Qseq_SS (65)	38	61	27	36	35		
RseqSal_SS (217)	66	32	211	70	52	28	
QseqSal_SS (961)	22	34	53	149	22	30	52
